# Elucidation of the Binding Orientation in α2,3- and α2,6-Linked Neu5Ac-Gal Epitopes toward a Hydrophilic Molecularly Imprinted Monolith

**DOI:** 10.1021/acsomega.3c06836

**Published:** 2023-11-06

**Authors:** Chau Minh Huynh, Liliia Mavliutova, Tobias Sparrman, Börje Sellergren, Knut Irgum

**Affiliations:** † Department of Chemistry, Umeå University, S-90187 Umeå, Sweden; ‡ Department of Biomedical Sciences, Faculty of Health and Society, Malmö University, SE-20506 Malmö, Sweden

## Abstract

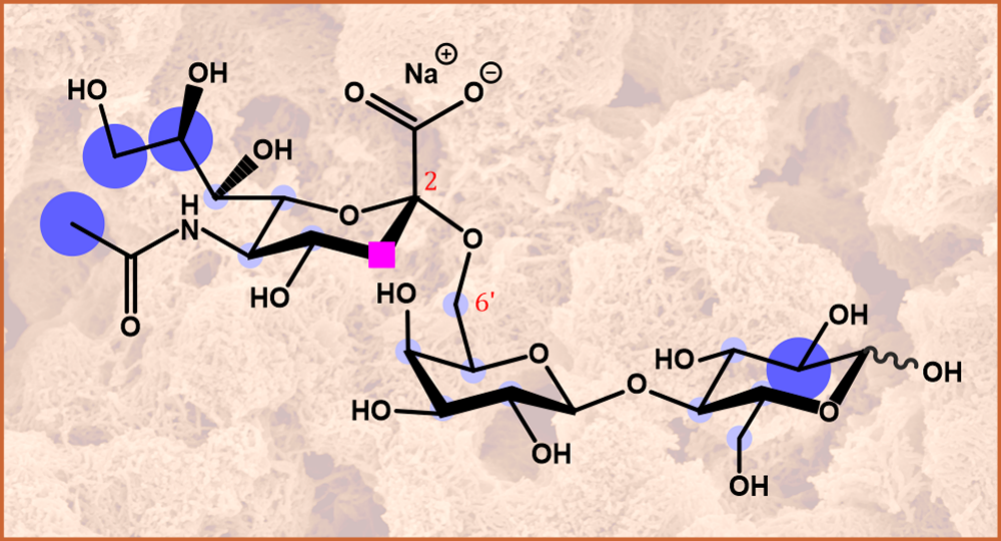

*N*-Acetylneuraminic acid and its α2,3/α2,6-glycosidic linkages with galactose (Neu5Ac-Gal) are major carbohydrate antigen epitopes expressed in various pathological processes, such as cancer, influenza, and SARS-CoV-2. We here report a strategy for the synthesis and binding investigation of molecularly imprinted polymers (MIPs) toward α2,3 and α2,6 conformations of Neu5Ac-Gal antigens. Hydrophilic imprinted monoliths were synthesized from melamine monomer in the presence of four different templates, namely, *N*-acetylneuraminic acid (Neu5Ac), *N*-acetylneuraminic acid methyl ester (Neu5Ac-M), 3′-sialyllactose (3SL), and 6′-sialyllactose (6SL), in a tertiary solvent mixture at temperatures varying from −20 to +80 °C. The MIPs prepared at cryotemperatures showed a preferential affinity for the α2,6 linkage sequence of 6SL, with an imprinting factor of 2.21, whereas the α2,3 linkage sequence of 3SL resulted in nonspecific binding to the polymer scaffold. The preferable affinity for the α2,6 conformation of Neu5Ac-Gal was evident also when challenged by a mixture of other mono- and disaccharides in an aqueous test mixture. The use of saturation transfer difference nuclear magnetic resonance (STD-NMR) on suspensions of crushed monoliths allowed for directional interactions between the α2,3/α2,6 linkage sequences on their corresponding MIPs to be revealed. The Neu5Ac epitope, containing acetyl and polyalcohol moieties, was the major contributor to the sequence recognition for Neu5Ac(α2,6)Gal(β1,4)Glc, whereas contributions from the Gal and Glc segments were substantially lower.

## Introduction

*N*-Acetylneuraminic acid (Neu5Ac) is the most common variant of the sialic acids, a class of alpha-keto acid sugars with nine-carbon backbones, generally found in the terminal positions of glycans attached to vertebrate cell surfaces. Attachment of sialic acid to a glucan signals to the cell that no more sugars should be added and the negative charge added on top of the underlying glucan structure provides a shield that significantly alters the properties of the cell.^1^ Sialic acids can form many kinds of glycosidic linkages to underlying sugar chains,^2−5^ providing sites for adhesive interactions. These are key features of cancer metastasis^6,7^ and terminal cancer cells with an aberrant glycan structure,^8^ as well as the receptors of viruses.^9,10^ Sialic acid-binding immunoglobulin-type lectins (Siglecs)^11,12^ on the cell surface bind sialic acids selectively, thanks to salt bridges that form between sugar residues and arginines at physiological pH^13,14^ when molecular configurations match.^15,16^ However, poor availability, high cost, and storage difficulty limit the use of Siglecs for glycan research development.^16^ Many synthetic glycan receptors are produced to overcome these impediments. Previous reports focus, *e.g.*, on elaborate three-dimensional clamshell constructs with strategically chosen hydrogen bonding abilities combined with a hydrophobic microenvironment to establish water-soluble glycan receptors.^17^ However, the unwieldy structure of such complex receptors limits the number of saccharides that can be targeted as well as their sizes.

Another approach to develop artificial glycan receptors is molecularly imprinted polymers (MIPs), based on establishing thermodynamic favorable complementary binding sites between a *template*, which can be the target molecule or a molecule featuring a characteristic epitope, and one or more functional monomer(s) polymerized in the presence of the template to form a cross-linked polymer matrix.^18−20^ Early MIPs were based on covalent boronic chemistry,^21^ which relied on the fast, stable, and reversible boronate ester bond formation between organic boronic acids and the diol functionalities on saccharide moieties.^22−25^ Another type of carbohydrate receptor uses polar and/or charged functional monomers^7,26^ to target both neutral mono/oligosaccharides^27^ and charged species.^7,28−31^ Sialic acids imprinted in bulk polymers yield MIPs with the intended affinity, but the conventional crush-and-sieve approach leaves a large proportion of the cavities locked in the interior of the polymer. This limits the analyte access and leads to heterogeneous binding affinities and slow binding kinetics.^32,33^ Other MIP technologies, such as surface imprinting^34−37^ tuned microenvironments,^38^ and the use of varying substrates^29,31,39−41^ have therefore been investigated and have proven their ability to address needs in glycomics.

Glycan imprinting has been extensively investigated only by bulk biophysical assays, which provide information about the overall binding affinity and capacity. However, knowledge of what parts of the target molecule actually contribute to the binding is equally interesting in the design of MIPs. A powerful tool for simultaneous study of reversible binding affinity and ligand-substrate interactions at the molecular level is saturation transfer difference nuclear magnetic resonance (STD-NMR) spectroscopy,^42−47^ which is based on the distance- and time-dependent transfer of saturation from protons of a saturated substrate to those of the ligand by the intermolecular nuclear Overhauser effect (NOE).^48^ This allows investigations of analyte binding toward their corresponding MIPs under realistic experimental conditions,^49,50^ as well as mapping of epitopes bound to affinity resins.^51−55^ In these applications, STD-NMR is used to identify the binding epitopes of low molecular weight ligands by mapping the protons that are in close contact with the substrate when the complex is formed.^56,57^ Protons of the released ligands that have been situated in the closest proximity to the receptor during a binding event show the highest STD effects and vice versa. However, the STD-NMR technique does not discriminate between specific and nonspecific binding,^42^ and the use of complementary methods is therefore needed to fully investigate the affinity of imprinted polymers.

In this work, we report on MIPs prepared in the presence of four different templates (Neu5Ac, Neu5Ac-M, 3SL, and 6SL), which showed recognition and selectivity toward predesigned sialic isoforms. Porous monolithic surface-imprinted polymers were prepared from the polar nitrogen-containing monomer melamine with formaldehyde as a cross-linker, via step-growth polymerization at four different temperatures, followed by characterization of the morphologies and the chemical compositions of MIPs and corresponding nonimprinted monoliths. STD-NMR combined with bound/free isotherm tests confirmed the key role of interactions between the Neu5Ac moieties of the test probes and the cross-linked melamine MIP, responsible for oriented binding of sialyllactose toward the MIP surface.

## Materials and Methods

### Reagents and Materials

1,3,5-Triazine-2,4,6-triamine (melamine; 99%), ammonium acetate (>98%), acetic acid (>99.7%), d-(+)-glucose (Glc; > 99.5%), d-(+)-galactose (Gal; > 99%), d-glucuronic acid (GA; > 98%), and d-lactose monohydrate (Lac; > 98%) were obtained from Sigma-Aldrich (Steinheim, Germany). *N*-Acetyl neuraminic acid (Neu5Ac, > 98%) was obtained from Santa Cruz Biotechnology (Dallas, TX, USA). Paraformaldehyde (extra pure) was purchased from BDH Chemicals (Poole, UK). Acetonitrile (ACN; analytical grade) and formic acid (FA; 98–100%) were obtained from Merck (Darmstadt, Germany). 3′-Sialyllactose sodium salt (3SL; > 98%) and 6′-sialyllactose sodium salt (6SL; > 98%) were produced by Carbosynth (Compton, UK). Methanol used for Soxhlet extraction was of analytical grade from Prolabo, obtained from VWR (Radnor, PA, USA). The Pluronic L61, an α,ω-hydroxy-poly(oxyethylene)-*block*-poly[oxy(1-methyl ethylene)]-*block*-poly(oxyethylene) triblock copolymer, (EO_2_PO_31_EO_2_; *M*_w_ ≈ 2000) used as porogen was obtained from BASF (Ludwigshafen, Germany). *N*-Acetylneuraminic acid methyl ester (Neu5Ac-M) was synthesized according to Rudrawar et al.^58^ and confirmed by NMR (Figure S1). Deuterated water (D_2_O; 99.9 atom-% D) and deuterated acetonitrile (CD_3_CN; > 99.8 atom-% D) were from Sigma-Aldrich.

### Preparation of Molecularly Imprinted Monoliths

A melamine-formaldehyde (MF) prepolymer was prepared by adding melamine (8.580 g, 67.8 mmol) and paraformaldehyde (6.000 g, 198 mmol) into a 100 mL round-bottom flask, followed by 48 mL water. This suspension was then immersed in an 80 °C preheated oil bath and magnetically stirred for 25 min, at which time the precondensate solution became essentially transparent. This MF precondensate solution was used immediately after cooling down to room temperature and was never stored for more than 4 h before use. A porogen solution was meanwhile prepared by dissolving Pluronic L61 (2.340 g) in 180 mL of acetonitrile. This porogen solution was stored at ambient conditions until spent and given a 30 s sonication in an ultrasonic bath before each withdrawal. To prepare the monomer cocktail, the templates (0.22 mmol each of Neu5Ac, Neu5Ac-OMe, 3SL, or 6SL to prepare MIPs designated as **M1**, **M2**, **M3**, and **M4**, respectively) were weighed into separate 20 mL screw cap glass vials, acting as molds, containing porogen solution (6.000 mL), followed by adding precondensate (7.800 mL). Formic acid (350 μL) was thereafter added as the polycondensation catalyst, followed by capping of the vials and vigorous mixing to form a homogeneous solution. The NIP (designated **N**) was prepared in the same way, but without a template added. The temperatures and reaction times investigated were −20 °C (96 h; freezer), 4 °C (24 h; refrigerator), 40 °C (24 h), and 80 °C (24 h). A Binder ED53 convective oven (Tuttlingen, Germany) was used for the experiments at 40 and 80 °C.

After the reaction, the monolithic materials were recovered by cracking the vials and subjected to Soxhlet extraction with methanol overnight, followed by drying in a vacuum oven at 40 °C overnight. The dry monoliths were crushed and then washed repeatedly with a 75:25% (v/v) mixture of 20 mM aqueous ammonium acetate and acetonitrile under sonication, using approximately 10 mL per gram. The supernatant solutions after being washed were collected and analyzed by LC-MS, as described below. These washing cycles were repeated, until no templates were detected in the supernatant. Finally, the materials were washed three times with methanol before drying in a vacuum oven at 40 °C overnight.

### Template Binding Test

Five milligram aliquots of materials were suspended and shaken in 1.000 mL of a template solution containing the templates 3SL or 6SL at concentrations varying from 50 to 1500 μM in a mixture of acetonitrile:water:formic acid, 43.4:56.5:0.1% (v/v) for 20 h at room temperature using an IKA Vibrax VXR orbital shaker (IKA-Werke, Staufen, Germany). The unbound templates in the supernatants were thereafter analyzed by the LC-MS method. The amount of bound analyte per unit surface area of polymer (*B*) was calculated according to
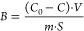
1where *C*_0_ and *C* are the analyte concentrations of the initial solution and supernatant, respectively, *V* is the total volume of the adsorption mixture, *m* is the mass, and *S* the specific surface area of the polymer, as measured by the Brunauer–Emmett–Teller (BET) method^59^ on a Tristar 3000 cryosorption instrument from Micromeritics (Norcross, GA, USA) with nitrogen as probe gas. The same instrument was also used to estimate the pore size and volume using the Barrett–Joyner–Halenda (BJH) scheme.^60^

Binding curves were established by plotting *B* against *C* and fitting the plots by nonlinear regression in OriginPro 8.5.1 (OriginLab, Northampton, MA, USA) to a Langmuir monosite model
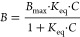
2where *B*_max_ is the maximum amount of probe bound to each surface area unit, and *K*_eq_ is the binding constant.

Imprinting factors (IF) were calculated by the saturated uptake ratios of the MIP and NIP, following
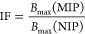
3

### Saccharide Binding Test

These tests were carried out in the same way as the template binding tests above, with the saccharides (Glc, Gal, GA, Lac, 3SL, or 6SL) at 1000 μM instead of the templates using the same solvent mixture. The percentage bound saccharide was calculated based on the initial probe concentration and the LC-MS analysis of the unbound probes in the supernatants.

### Instrumentation and Conditions for the LC-MS Analysis

LC-MS analyses of the templates and saccharide probes in the binding experiment supernatants were carried out by a Surveyor-LCQ Fleet Ion Trap LC/MS^*n*^ instrument from Thermo Scientific (Waltham, MA, USA). A 100 mm long by 4.6 mm i.d. PolySULFOETHYL A column (PolyLC, Columbia, MD) with a 200 Å pore size was used for separation in HILIC mode. The mobile phase contained 75% acetonitrile and 25% 20 mM aqueous ammonium formate by volume, delivered at a 0.5 mL/min flow rate. The ESI capillary temperature was 325 °C with a sheath gas flow at 60 AU. MS detection was operated in negative mode with source voltage and current of 4 kV and 100 μA, respectively. Other parameters were tuned automatically. The working ranges were 20–1000 μM for all probes, with 10 μL injection volume. MS spectra were acquired in the *m*/*z* range 50–1000. Extracted ion chromatograms of monoisotopic *m*/*z* values for the respective compounds were obtained using Xcalibur 2.5.5 SP1 software from Thermo Scientific. The *m*/*z* list is shown in Table S1.

### Sample Preparation for STD-NMR

Crushed dry monolithic material was extensively washed with 43.5% (v/v) aqueous acetonitrile and then with methanol, followed by drying at 40 °C under a vacuum for 48 h. Test solutions were prepared containing 12 mg/mL of a sialyllactose probe (3SL or 6SL) in the same solvent mixture used in the binding test experiments. Five hundred microliter aliquots of test solution containing each of the probes (3SL or 6SL) were equilibrated with 35 mg of each adsorbent (**N** or **M4**) in 1.5 mL Eppendorf tubes. These tubes were then capped and shaken for 20 h at room temperature on a Vibrax VXR orbital shaker. The adsorbent suspensions thus obtained were transferred into disposable NMR rotor inserts and packed under centrifugal force. The inserts were thereafter closed and immediately fitted into 4 mm ZrO_2_ rotors with caps.

### Investigating the Interaction between Sialyllactose and Imprinted Monolith by STD-NMR

All suspended-state STD-NMR spectra were recorded on a Bruker Avance III 500-MHz NMR spectrometer at a spinning rate of 4.2 kHz using 4 mm ZrO_2_ rotors. A total of 2400 transients were recorded in each experiment. The saturation was done by a Gaussian enveloped pulse train during 2 s (50 ms pulses looped l5 = d20/50 ms = 2000/50 = 40 times, at peak power 0.1 W) with saturation frequencies according to the Fq2 list (1800, 2550, and 12600 Hz). This was followed by the 90 deg excitation pulse and the CPMG pulse train for the T_2_-filter^61,62^ with L6 + 2 = 22 times chem shift echoes [200 μs–180° pulse −200 μs], before acquisition. The “delta1” relaxation delay had been d1-d20 = 3–2 s = 1s before starting the next train of saturation pulses. The number of blocks (3) in the Fq2 list was interleaved in an inner loop before the number of scans, followed by subtraction of saturated spectra from reference spectra to obtain the STD-NMR.^63−65^ The STD responses, *a.k.a.* transfer efficiencies, were calculated according to the equation as the ratios of the intensities of the signals in the STD-NMR spectrum (*I*_STD_) and the signal intensities of the corresponding reference spectrum (*I*_0_).
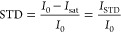
4

The STD of each experiment was normalized to the STD of the H1 peak (at ∼5.4 ppm) of the glucose moiety. The STD difference between the NIP and the MIP of each probe was calculated according to the equation
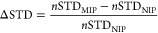
5where *n*STD is the STD of the MIP and NIP hydrogens, normalized against the H1 peak of the glucose moiety.

### Material Characterizations

See Supporting Information for descriptions of the characterization experiments.

## Results and Discussion

### Effect of Polymerization Temperature and Time on the Cross-Linked Melamine-Based Monolith Formation

Cross-linked melamine, utilized as a hydrophilic scaffold for MIPs,^66−68^ was prepared by step-growth polymerization of melamine and paraformaldehyde in a tertiary porogen mixture adapted from our previous communication,^66^ with some modification as described in the experimental section. Polycondensation between melamine and formaldehyde occurs in the presence of H^+^ or OH^–^ as catalyst and is promoted by temperature.^69,70^ However, increasing temperature weakens the interactions between the template and the growing polymer due to the temperature-dependent strengths of hydrogen bonds and permanent dipole-permanent dipole interactions (Keesom forces), which are essential for molecular imprinting of polar templates. The MF prepolymer predominantly consists of melamine or oligomers with varying degrees of methylolation, on average close to 3 in these experiments. Upon activation with an acidic catalyst, electrophilic imine cations are formed, which trigger the polycondensation.^71^ This reactivity means that good nucleophilic groups of the template molecules capable of being involved in the polycondensation may need to be protected before polymerization.^72−75^ Excessive covalent incorporation of templates could otherwise lead to lower capacity and even loss of template recognition after the finished synthesis. Although the templates chosen (Figure 2) do not contain any particularly good nucleophilic groups, we chose in these syntheses to omit a final polymerization step, which is common in MF polymerizations. Moreover, imprinted sites are created by all moieties present in the polymer cocktail, including porogen solvents,^76^ which can lead to nonspecific binding sites^77^ or difficulties in template removal.^78,79^ We therefore chose acetonitrile as a non-hydrogen-bonding low molecular weight solvent of low reactivity. As a catalyst for the reaction, we selected formic acid, the smallest possible carboxylic acid. Moreover, most of the interesting targets for creation of “synthetic antibodies” are naturally hydrophilic compounds with tertiary structure,^80,81^ such as peptides, protein, and glycans, which are easily denatured by heat.^82−85^ If a large template had been chosen as the template for imprinting, it could therefore have a spatial constellation quite different compared to the native molecule in its natural environment, leading to inferior binding affinity. Four small molecule templates were therefore chosen in an attempt to establish selectivity for the terminal sialic groups of Neu5Ac-Gal antigens; Neu5Ac and its methyl ester and Neu5Ac linked to lactose through the 3- and 6-hydroxy groups of its galactose moiety.

**Figure 1:**
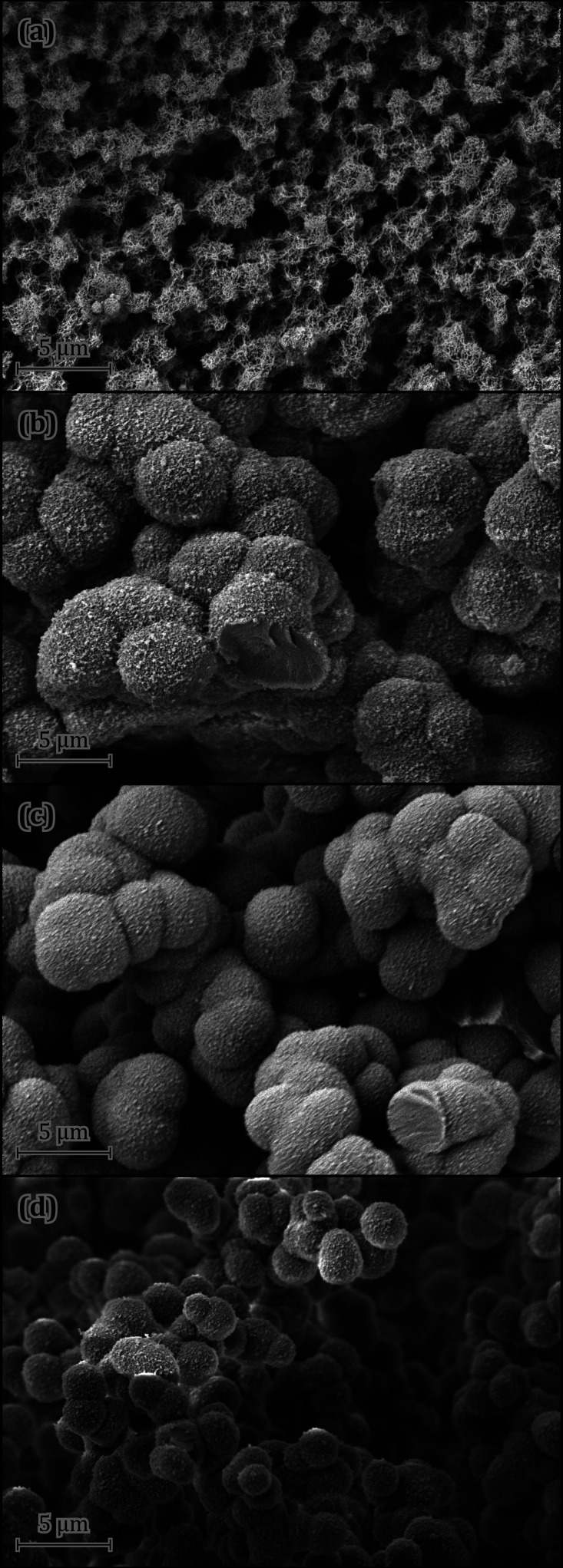
Field emission scanning electron micrographs at 10,000× magnification of random fracture cross-section surfaces of NIP monoliths polymerized at (a) – 20 °C, (b) 4 °C, (c) 40 °C, and (d) 80 °C. Contrast has been adjusted to represent the full 8-bit greyscale.

The strength of monomer–template complexes is known to decrease with increasing polymerization temperature due to faster molecular dynamics.^86−89^ In this work, we therefore chose a wide temperature range (−20, 4, 40, and 80 °C) for the initial experiments of the acid-catalyzed polycondensation of the MF precondensate. The SEM micrographs of the resulting polymers in Figure 1 show that all polymerization temperatures except −20 °C yielded fused globe-like structures, characteristic of nucleation and growth as phase separation mechanism.^90^ The domain sizes varied inversely with the polymerization temperatures: 5.0 ± 0.39, 4.1 ± 0.54, and 2.4 ± 0.33 μm for 4, 40, and 80 °C, respectively (Figure 1b–d). However, only the sample polymerized at 80 °C formed a space-filling monolith as in our previous work,^66^ whereas the other nonfreezing conditions (4 and 40 °C) led to monolithic materials that did no fill the glass vial molds entirely but underwent syneresis with ≈20 vol % expelled liquid. Despite the expected imprinting enhancement at low temperatures, it must also be noted that the rates and yields of the polymerizations and also the structures were affected by temperature in these three nonfrozen polymerization attempts.

Fortunately, the cryopolymerizations at −20 °C produced solid, space-filling, porous monolithic polymers without syneresis, showing a radically different morphology (Figure 1a). At higher magnification, these MF cryo-monoliths showed a bimodal pore morphology with micrometer-sized through-pores formed by the acetonitrile-rich phase, and an intraskeleton porosity of mesopores and small macropores with connected-rod features (Figures S2a and S3), characteristic of sol–gel synthesized polymers formed in mixtures undergoing spinodal decomposition.^91^ The solvent mixture in the polymerization step contains 43.5% acetonitrile in water and has an upper critical solution temperature (UCST) of −2 °C.^92^ At temperatures above UCST, the mixtures form what macroscopically appears to be single phases,^93^ but it is well-known that these mixtures are microscopically heterogeneous in most ratios at ambient temperatures.^94^ As the temperature decreases below the UCST, the solvent mixture used in the polymerization consequently undergoes a phase segregation, forming two phases of mutual solubility by spinodal decomposition.^95−98^

The presence of prepolymer and porogen will most certainly affect this ideal phase separation behavior, as the water solubility of the prepolymer, best represented as trimethylolmelamine, should be high due to its calculated octanol–water partitioning coefficient log *K*_OW_ of −0.83.^99^ The block copolymeric porogen is also expected to span and stabilize the interface between the water-monomer and acetonitrile phases. The polymer resulting from the acid-catalyzed cross-linking of the methylolmelamine prepolymer^100,101^ should moreover have a high affinity for the aqueous-rich phase (log *P* = −0.8, estimated by Pu-Chem/X log *P*3 3.0) and is therefore going to be confined to the water-rich phase, as an intersecting pore system is created in the space occupied by the acetonitrile-rich phase. In the cryopolymerization experiment, the precursor cocktail appeared to be frozen after about 30 min at −20 °C and the polymerization took place to form a cryogel,^102^ and the polymerization kinetics was still reasonably fast due to the freeze concentration^103^ of the monomer system. This freeze–thaw process^104,105^ produced mesoporous polymer nanofiber networks (Figure 1a) templated by the frozen state of parts of the precursor mixture between the growing polymer chains, structures that remained as the samples were thawed at room temperature.

The morphologies of the cryopolymerized monoliths depended strongly on the freezing rate. When the monomer cocktail was slowly frozen in the freezer at −20 °C for 96 h, the SEM images showed that the structures created were essentially anisotropic (Figure S2a). However, when the mixture was subjected to flash-freezing (30 s submersion in liquid nitrogen at −196 °C, followed by 96 h polymerization at −20 °C), directional freezing^106−108^ resulted in monolithic materials with clearly isotropic structures (Figure S2b). Slow freezing was chosen to produce an anisotropic scaffold, as it was deemed to be more suitable for the imprinting process and for the evaluation experiments. Since initial attempts to prepare molecularly imprinted polymers for 3SL and 6SL by polymerization at 80 °C resulted in MIPs without imprinting effects (data not shown), we chose −20 °C for 96 h as the synthesis conditions for further investigations.

### Design and Synthesis of MIPs

**Figure 2:**
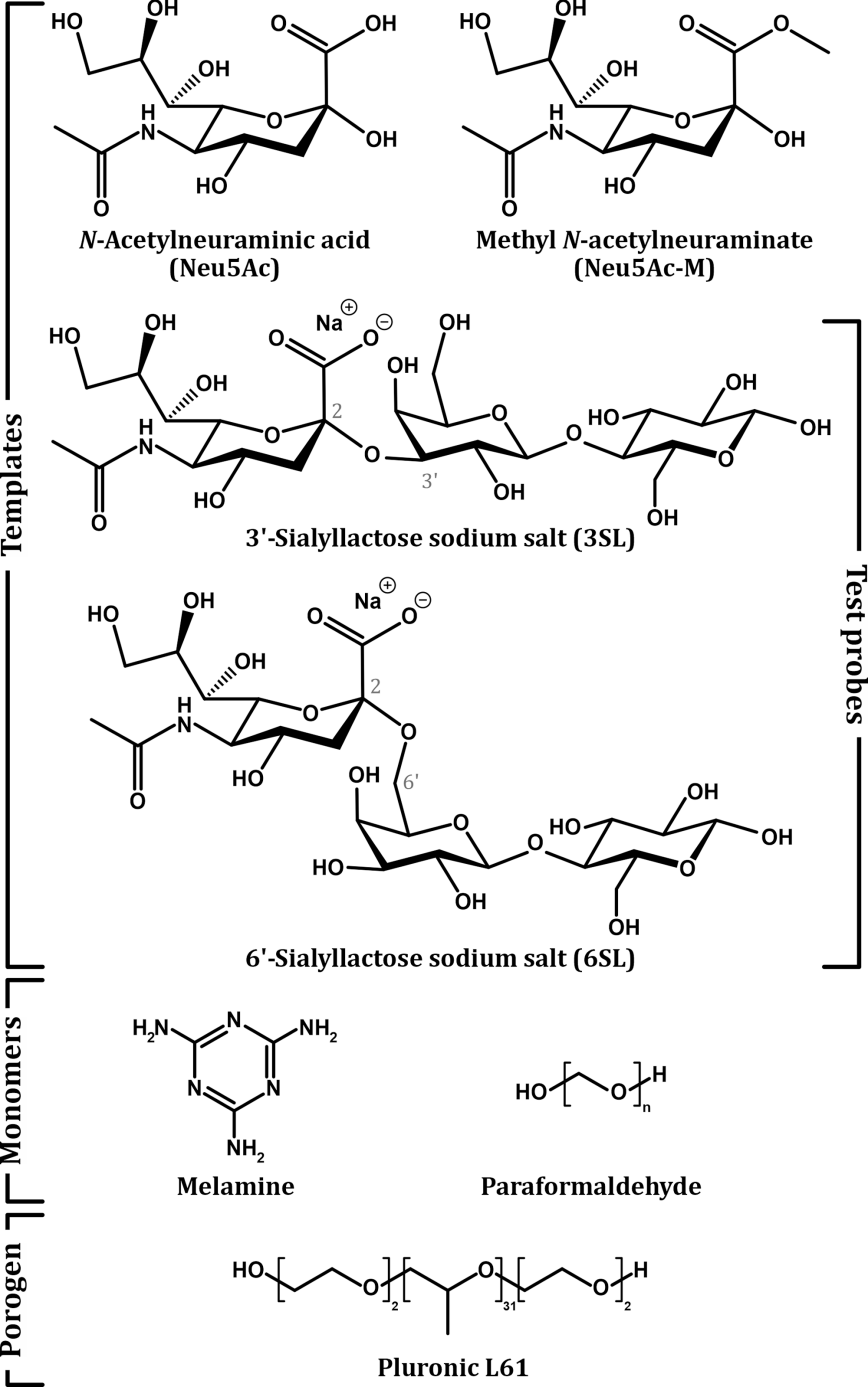
Chemical structures of the four templates (Neu5Ac, Neu5Ac-M, 3SL, and 6SL), the two monomers, and the porogen. The templates also act as probes for the imprinted monoliths toward the sialic acid moiety, with the sialyllactoses (3SL and 6SL) aimed at determining the selectivity for the α2,3 and α2,6 linkage conformations.

Our recent report on a partly aqueous biphasic step-growth polymerization^66^ has shown that melamine-based monoliths have preferred binding toward hydrophilic compounds with negative charge, thanks to electrostatic interaction, hydrogen bonding, and low propensity for π–π interactions.^109^ We here adopted the melamine-based approach for the preparation of monolithic molecularly imprinted polymers for the Neu5Ac moiety and its glycosidic linkage conformations Neu5Ac(α2–3)Gal and Neu5Ac(α2–6)Gal, using lactose-linked compounds 3′- and 6′-sialyllactose (3SL and 6SL) as model molecules. The *N*-acetylneuraminic acid (Neu5Ac) moiety was used as a template because of its demonstrated ability of forming molecular imprints.^32,36,110,111^ Previous studies on Siglecs have revealed that the binding of sialic acid takes place in a shallow pocket, thanks to the formation of a salt bridge between the negatively charged carboxyl group of sialic acid and an arginine residue in the Siglec.^11,14^ In this work, we used four different templates to prepare imprinted materials, Neu5Ac, Neu5Ac-M, 3SL, and 6SL, which all contain the Neu5Ac epitope intended to mimic the sialic acid antigen terminal. These MIPs are henceforth referred to as **M1**, **M2**, **M3**, and **M4**, respectively (Figure 2). In the case of **M2** (Neu5Ac-M as the template), the carboxylic group had been blocked by forming a methyl ester to investigate the role of the carboxylic acid group in the imprinting process. The comparison between **M3** and **M4** (3SL and 6SL as templates, respectively) was used to evaluate the affinity and discrimination of imprinted materials between sialic linkage modes to the galactose subunit of lactose.

The nonimprinted (**N**) and imprinted (**M1–M4**) monolithic products were characterized by nitrogen cryo-sorption and FE-SEM to evaluate their porosities and morphologies, and by FT-IR, ^1^H NMR, and ^13^C CP-MAS NMR for confirming their chemical composition. For space reasons, the results from these characterizations are shown in the Supporting Information, along with the accompanying experimental descriptions. References to Figures and Tables enumerated by an initial “S” refers to data located there.

FE-SEM images (Figure S3) and BJH analysis^60^ of nitrogen cryo-sorption data (Figure S4) showed that the skeletons of the monoliths polymerized in the frozen state consisted of nanofibrillar mesoporous polymer networks with nominal pore diameters ranging from 10 to 50 nm. These mesoporous skeletons are transected by 2–3 μm macropores, establishing hydraulic flow pathways created by a continuous acetonitrile-rich domain, as the mesoporous polymer formed in a predominantly aqueous phase. The morphologies of these monoliths differed from the templates used. Monoliths **N** and **M4** had higher specific surface areas, 152 and 108 m^2^/g, as measured by the BET method, respectively, in comparison with the three other MIPs (46–66 m^2^/g). The median mesopore diameters of **N** and **M4** (approximately 28 nm) were also smaller than **M1**–**M3** (approximately 40 nm)(Figure S4). Porosity distributions were calculated in the standard manner by using the BJH method^60^ of nitrogen cryo-sorption measurement, in which significant differences were observed (Figure S4). Monoliths named **N**, **M2–M4** showed a similar pattern of mesopore volume distributions with more than 50% of their pore volume contributed by >20 nm pores. On the contrary, only 36% of the mesopore volume of monolith **M1** was provided by >20 nm pores and the mesopores were relatively uniformly distributed in the range of 2–50 nm. This observation also agreed with the FE-SEM images of **M1** and **M2** (Figure S3) where their thicker fiber diameters (Table S2) seem to have resulted in overall denser structures compared to the other monoliths.

The FE-SEM micrographs also revealed differences in nanofiber sizes among materials, as the nanofiber diameters of **M1** and **M2** (125 and 123 nm) were significantly larger than that of **N** (36 nm) (Table S2), whereas **M3** and **M4** showed intermediate nanofiber diameters (59 and 68 nm). We see two plausible reasons for this. First, the template-monomer complex can effect the polarity of growing polymer chains, affecting the precipitation rate, backbone size, and porosity.^7,112^ Second, reactions between the MF prepolymer and the plentiful hydroxyl groups of the templates during the imprinting step cannot be completely ruled out. Yet in a study investigating cross-linking of MF prepolymer with hydroxylated acrylic resins, Bauer and Dickie^113^ did not detect any cross-linking reactions at curing temperatures of 90 °C and below. In a more recent study, Kohlmayr et al.^114^ attempted to use glycerol, sucrose, and starch as polyols to modify MF resins and found that only glycerol could be covalently reacted with methylolated melamine. The risk of template hydroxyl groups reacting with the MF prepolymer should therefore be nonexistent at the cryotemperature during the polymerization step. The FTIR and the solid-state ^13^C NMR spectra of the monoliths were highly similar (Figures S5 and S6), and there were no noticeable differences in the FTIR spectra for bands assigned to –OH and C–H, which are the main functional groups of the templates. The CP–MAS ^13^C NMR spectra also confirmed the presence of both methylene and ether bridges on the final products. No signals were present that could emanate from incorporation of templates, and we therefore conclude that the differences in morphologies were caused by interactions between the templates and the monomer phase during the step-growth polymerization.

### Evaluation of Affinity and Selectivity of the Imprinted Monoliths

**Figure 3:**
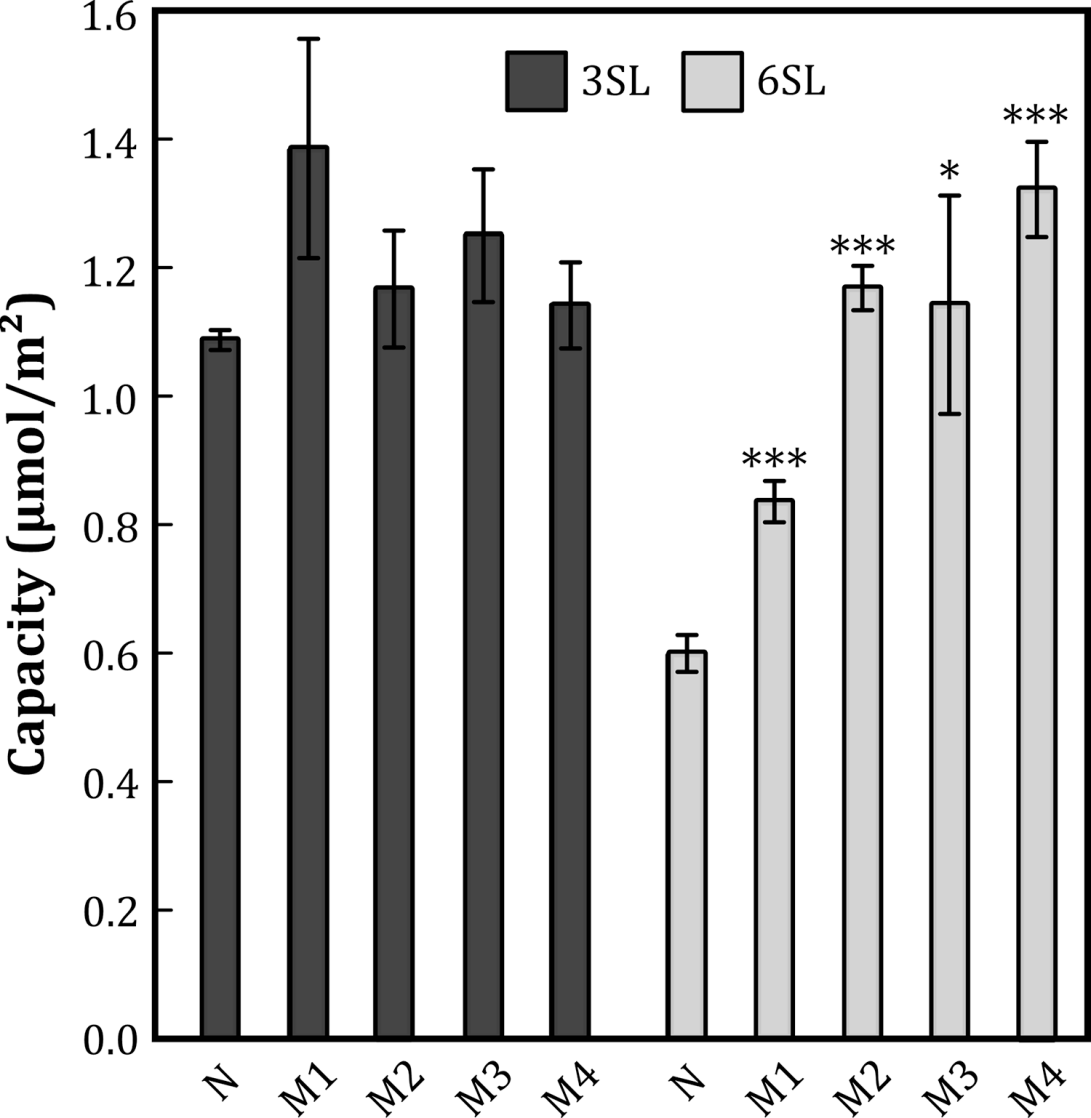
Binding capacities from binding isotherms of 3SL and 6SL with four imprinted monoliths and NIP. Asterisks indicate significant differences between nonimprinted (**N**) and imprinted monoliths (**M1–M4**) (*t*-test; *p < 0.05, **p < 0.01, and ***p < 0.001).

The affinities of the imprinted monoliths for the sialic epitope were assessed by template rebinding using the bound-free isotherm method^115^ on crushed monoliths, using 3SL and 6SL as probes dissolved in the same solvent mixture as used in the polymerization. The results of these experiments are shown in Figure 3 and Table S3. All monoliths (**N**, **M1–M4**) adsorbed 3SL with similar capacities and imprinting factors (IFs) in the range of 1.05–1.27 (Figure 3), indicating that the imprinting efforts had no influence on the rebinding of 3SL, regardless of which templates had been used. With 6SL as a probe, the pattern was quite different, with significant imprinting affinity seen for all templates. A simplistic explanation to this difference could be that the 2,6-bonded trisaccharide 6SL assumes a more “kinked” structure (Figure S7) in comparison to the more extended structure of 2,3-bonded 3SL, which will be flexible enough to access more nonspecific or induced binding sites.^116,117^ Another observation is that the binding capacity of the nonimprinted material toward 3SL was 1.8 times higher than for 6SL (Table S3), which also affected the difference in imprinting factors. This agrees with findings of Singh et al. concerning binding of 3SL and 6SL toward TAdV-3 fiber head.^118^ The binding orientations of 3SL and 6SL on the imprinted monoliths were further evaluated by STD-NMR spectroscopy, as discussed below. The material **M1**, imprinted using Neu5Ac as template, showed a selectivity for 6SL (IF = 1.39), in line with previous research on methacrylate-based scaffolds.^32,110,111^ Materials **M3** and **M4**, imprinted using 3SL and 6SL as templates, both showed significant selectivity toward 6SL, with imprinting factors 1.91 and 2.21, respectively. Their higher affinities for 6SL could be caused by several factors, one being differences in template conformations. Neu5Ac exists as α- and β-anomers, whereas the α-anomer is the only conformation of sialic acid bound to glycans, also in sialyllactose. In aqueous solution, Neu5Ac exists mainly in the β-anomeric form (95%),^119,120^ as confirmed by ^1^H NMR in Table S4. Spatial dissimilarities between the templates and the target analytes caused by anomerization are therefore likely to reduce the imprinting affinity when Neu5Ac is used as a template for terminal Neu5Ac linked by *O*-glucosidic bonds. Another factor could be the more complex structures of the sialyllactose templates used to prepare **M3** and **M4**, compared to Neu5Ac, which could have produced surface interaction sites with better orientation for 6SL rebinding.^121,122^ Moreover, the morphological differences among the monoliths accounted for above hint at an effect of the templates on the surface chemistries during the polymerization step, and many imprinted sites formed by the monosaccharide Neu5Ac could be located where the trisaccharide probes 3SL and 6SL do not have access.^123^

The motivation for including Neu5Ac-M as a template for **M2** was to evaluate the role of the sialic carboxylic acid in the imprinting process, in addition to testing the influence of anomerization. The ^1^H NMR spectrum of H3 of Neu5Ac-M (Table S4) confirmed that like Neu5Ac, Neu5Ac-M also existed as 95% β-anomer. The capacity of monolith **M2** for binding of 6SL was significantly higher than that of **M1**, 1.17 ± 0.069, and 0.84 ± 0.064 μmol/m^2^, respectively (Table S3). As mentioned above, FTIR and CP-MAS ^13^C solid-state NMR (Figures S5 and S6) showed that the chemical compositions of **M1** and **M2** were practically identical, whereas their morphologies differed remarkably. Despite equal nanofiber diameters (125 and 127 nm, Table S2), the pore volume distribution of **M2** was the same as **M3** and **M4**, with 50% of the mesopores larger than 20 nm.

The selectivities of these MIPs were evaluated by batch binding isotherm assays with glucose (Glc), glucuronic acid (GA), galactose (Gal), lactose (Lac), 3′-sialyllactose (3SL), and 6′-sialyllactose (6SL) as model saccharides. The MIPs were incubated in the same mixture of acetonitrile, water, and formic acid used in the polymerization step before determining the fractions bound by LC-MS, as described in the Supporting Information.

**Figure 4:**
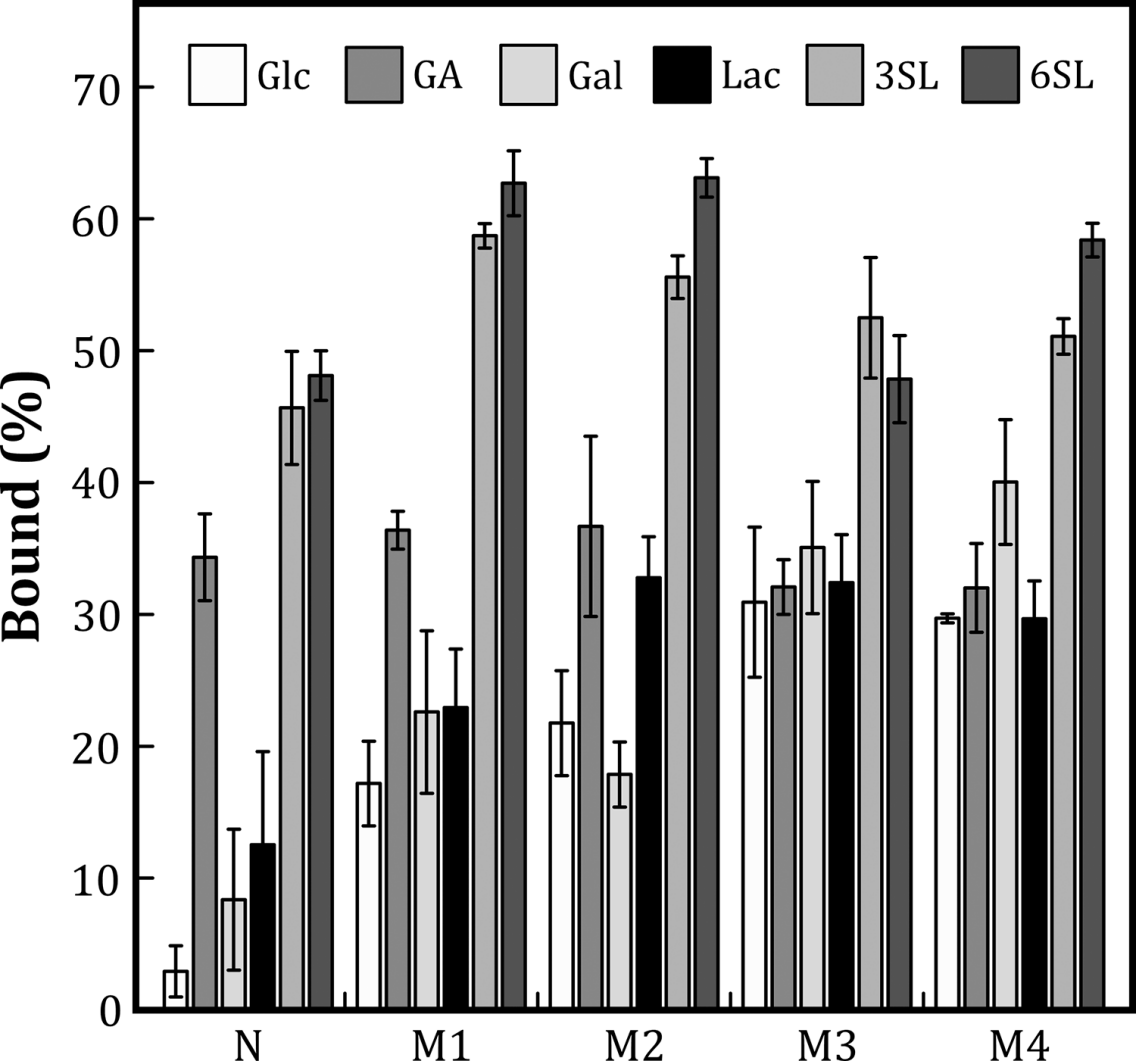
Binding percentages of several saccharides on the nonimprinted and four imprinted monoliths.

As expected, the binding of 3SL and 6SL onto all imprinted monoliths were significantly higher than the nonsialic monosaccharides (Glc, Gal, and GA) and disaccharides (Lac), with binding percentages amounting to ≈60% of the initial probe amounts. About 20% of Glc, Gal, and Lac were bound onto **M1** and **M2,** which was lower than their bound percentages on **M3** and **M4** (30–40%) (Figure 4). It should be noted that the sialyllactose-based templates of monoliths **M3** and **M4** were composed of the monosaccharides Neu5Ac, Gal, and Glc in that sequence and could hence have created sites with enhanced binding efficiency for these moieties. A probe breaking the patterns of other nonsialic probes was GA, which had equal affinity for all the imprinted monoliths with ≈35% binding. This could be due to the positive charge surface of the melamine-based structure,^66,124−126^ which could offer an electrostatic interaction to the carboxylic groups of GA and Neu5Ac.

In summary, the monoliths did not show any specificity toward 3SL (α2,3 linkage of Neu5Ac-Gal), whereas imprinted materials with affinities for 6SL (having an α2,6 linkage of Neu5Ac-Gal) had been created in monoliths **M1–M4** by four different templates with imprinting factors 1.39, 1.95, 1.90, and 2.21, respectively.

### Binding Orientation Investigation by STD-NMR

Solid-state CP-MAS ^13^C NMR spectra of the nonimprinted (**N**) and imprinted (**M1–M4**) monoliths (Figure S6) verified high chemical composition similarities and proved that the imprinting templates were not covalently incorporated in the MF scaffold. The intense peaks at 166.35 ppm from the triazine ring carbons are characteristic of polymers that are composed of melamine rings, with additional signals from the covalently methylene bridge cross-links (−N–**CH**_**2**_–N–; two peaks at 48.78 and 54.63 ppm), as well as ether bridges and methylol groups (−N–**CH**_**2**_–O–**CH**_**2**_–N– and/or – N–**CH**_**2**_–OH, at 65.51 and 72.34 ppm). Moreover, their physical morphologies were also similar, as were their specific surface areas and porosities (Figures S3 and S4). Differences observed between the **N** and the **M4**, chosen because of their desired morphologies and similar capacities for 3SL and 6SL, in the abovementioned rebinding affinity and the following STD-NMR measurements must therefore be due to differences in surface conformations due to the imprinting process.

**Figure 5:**
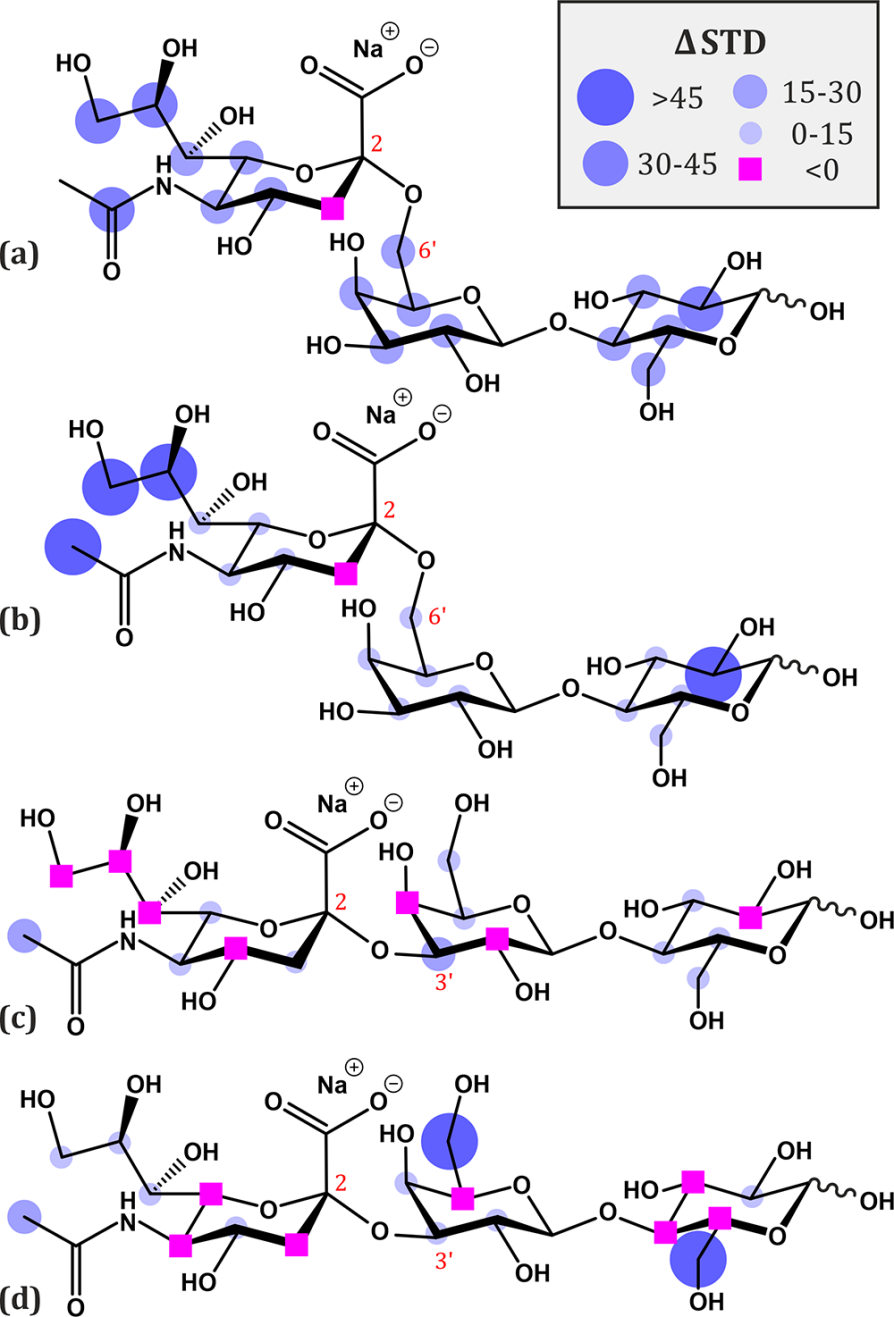
Relative differences in STD effect as percentages for (a, b) 6′-sialyllactose and (c, d) 3′-sialyllactose bound to imprinted versus nonimprinted monoliths. The irradiation frequencies were (a, c) 1800 Hz (3.6 ppm) and (b, d) 2550 Hz (5.1 ppm).

To elucidate the specific binding site and orientation of analyte molecules in more detail, monoliths were equilibrated with 3SL and 6SL and selectively saturated at 3.60 ppm (1800 Hz) and 5.10 ppm (2550 Hz), with the off-resonance frequency at 25.2 ppm (12600 Hz) in the nonsignal region. Protons of 3SL and 6SL, which under these experimental conditions closely interact with the magnetically saturated polymer backbones, will receive magnetization through the NOE.^127^ The saturation transfer (STD) signals resulting from this contact were calculated as described in the experimental section (eq 4) and normalized based on anomeric proton STD response of the glucose moiety, which does not contribute to the binding of sialyllactose toward the imprinted receptor.^46,128,129^ The differences in STD (Δ*S*TD) between nonimprinted (**N**) and imprinted (**M4**) monoliths were then quantified from normalized STD signals according to eq 5. The proton peaks of the sialyllactose probes were identified by ^1^H NMR measurements (Figures S8 and S9) and compared with previous studies.^46,48,130^ The Δ*S*TDs are illustrated by the dotted heat map in Figure 5.

When 3SL was used as a probe (Figure 5c), none of the STD signal changes were significant, defined as less than 15% signal increase. On several protons, the STD signals of the NIP monolith were actually *larger* than on the MIP, indicated as magenta squares in Figure 5 and negative STD difference values in Table S5. This is in agreement with the rebinding results showing similar binding capacities of 3SL toward the **N** and **M4** monoliths. Adsorption of 3′-sialyllactose toward hydrophilic melamine-based monoliths was hence nonspecific and all parts of the molecule appeared to contribute equally to the nonspecific interactions.

When the 6SL was used as a probe (Figure 5a), a significantly divergent pattern was revealed. Thanks to the imprinting effect, which was evaluated above by the bound-free isotherm method (Figure 3), the STDs were enhanced for all protons of the 6SL molecule, especially for the Neu5Ac epitope. Protons of the acetyl group and polyalcohol chain of the imprinted monolith **M4** showed a 32% increase in the STD signal. Protons of the other moieties, Glc and Gal, also had positive values of STD differences, by approximately 20% (Table S6). This significant saturation transfer confirmed the presence of binding sites for 6′-sialyllactose in the 6SL-imprinted monolithic cross-linked melamine polymer **M4**.

Moreover, the excitation frequency plays an important role in ligand–receptor mapping by STD-NMR.^46^ The monoliths, prepared from melamine and paraformaldehyde (Figure 2), contain a triazine ring cross-linked by methylene and ether bridges.^66^ The only “stable” (nonexchangeable) protons of the polymerized monoliths are those of the methylene bridges between amine nitrogens of the melamine molecules, or between a melamine amine and an oxygen atom originating from paraformaldehyde. The solid-state proton NMR spectra of the cross-linked melamine monoliths therefore showed a single broad peak centered around 3.40 ppm (Figure S10). Protons of amines (primary and secondary) and hydroxyl groups are labile due to fast exchange in the presence of protic solvents like water and will not participate in saturation transfer from the monolith. Based on this insight, we chose to saturate the monolith structures at shifts corresponding to 90 and 10% of the maximum proton intensity, at 3.60 and 5.10 ppm, respectively.

The dot heat map of STD differences was also constructed for two different excitation frequencies to investigate whether this caused dissimilarities in the saturation transfer patterns. In the case of 3SL with the pulse at 5.10 ppm (Figure 5d), on the downfield shoulder of the broadened absorption methylene proton peak of the monolith matrices, changes in STD difference can be seen for some protons, but the main results were in the agreement with the transfer difference recorded after saturation at 3.60 ppm, i.e., on neither material **N** nor **M4** had 3SL interacted with a closeness and duration sufficient to cause significant saturation transfer. Nevertheless, for 6SL with an excitation pulse at 5.10 ppm (Figure 5b), most proton STD signals, except for Neu5Ac–H3, showed a positive difference between **N** and **M4** monoliths. The relative magnetization transfers were above 45% for protons in the acetyl and polyalcohol (C7–C9) groups of the Neu5Ac epitope. From Figure 5b, it could appear as if the Glc-H2 proton had received significant saturation transfer, but its spectrum overlaps with the strongly affected Neu5Ac–H8 and Neu5Ac–H9 protons. All other protons showed positive Δ*S*TDs, although their magnitudes were <15% (Table S6). The reason for the differences between these two excitation frequencies could be the difference in the magnetization efficiency of the monolithic substrates. For the experiments at 5.10 ppm, the charged magnetization would be only ≈10% compared to the excitation at 3.60 ppm and the expected saturation transfer should therefore be more efficient between closer atoms than between atoms further apart. The significant Δ*S*TD signals of the Neu5Ac–H8, Neu5Ac–H9, and Neu5Ac–Ac protons (Table S6 and Figure 5a,b) provide a strong evidence for the selective binding of the 6′-sialyllactose molecule toward the imprinted monolith **M4** with the Neu5Ac epitope orientated toward the binding site.

## Conclusions

Attempts were made to synthesize imprinted hydrophilic monoliths with selectivity toward 3′- and 6′-sialyllactose from hydroxymethylated melamine using four different templates, namely, Neu5Ac, Neu5Ac-M, 3SL, and 6SL. The affinity and selectivity of the MIPs were verified by the bound/free isotherm method fitted to the monosite Langmuir model. Imprinting effects were seen with 6′-sialyllactose as a test probe, whereas MIPs selective toward 3′-sialyllactose were not successfully produced, most likely because of highly nonspecific adsorption of this negatively charged trisaccharide to the highly polar and positively charged MF material surfaces. The bound percentages of targets, 3′- and 6′-sialyllactose, were significantly higher than other saccharides, such as glucose, galactose, glucuronic acid, and lactose. The directional interaction between 6′-sialyllactose on its imprinted monolith was revealed by the STD-NMR measurements, where the selective affinity appeared to be caused by preferential interaction of the Neu5Ac moiety and the hydrophilic melamine-based scaffold. Limitations of the current study could be the relatively low imprinting factors and lack of evaluation of the monolithic MIPs with biological samples; this is beyond the scope of this article.

## Supporting Information Available

Additional reagents, experimental details, background information, and results for the characterization experiments (PDF)
