# Supplementary material for: Elucidation of the Binding Orientation in α2,3- and α2,6-Linked Neu5Ac-Gal Epitopes toward a Hydrophilic Molecularly Imprinted Monolith
Source: ACS Omega. 2023 Nov 6;8(46):44238–49. doi: 10.1021/acsomega.3c06836 (PMC10666243; doi:10.1021/acsomega.3c06836)
Supplement: Supplementary file 1 — ao3c06836_si_001.pdf [file ao3c06836_si_001.pdf]

Supporting Information

Elucidation of the Binding Orientation in α2,3- and α2,6-Linked Neu5Ac-Gal Epitopes towards a Hydrophilic Molecularly Imprinted Monolith

Chau Minh Huynh[a], Liliia Mavliutova[b], Tobias Sparrman[a], Börje Sellergren[b], Knut Irgum[a*]

a) Umeå University, Department of Chemistry, S-901 87 Umeå, Sweden.

b) Malmö University, Faculty of Health and Society, Department of Biomedical Science, S-205 06 Malmö, Sweden.

Table of Contents

Characterization and Evaluation Procedures S-2
Field-Emission Scanning Electron Microscopy S-2
Nitrogen Cryosorption S-2
Diffuse Reflectance Fourier Transform Infrared Spectroscopy S-2
Liquid Nuclear Magnetic Resonance Spectroscopy S-2
Solid-state Nuclear Magnetic Resonance Spectroscopy S-2
Geometry structure optimization S-3
Figure S1. ^{1}H and ^{13}C NMR spectra of *N*-Acetylneuraminic acid methyl ester (Neu5Ac-M). S-2
Figure S2. FE-SEM micrographs of NIP monoliths prepared by different freezing schemes S-2
Figure S3. FE-SEM micrographs of non-imprinted (**N**) and imprinted (**M1-M4**) monoliths S-3
Figure S4. BJH desorption dV/dlog(D) pore volume plots of the monoliths S-3
Figure S5. Fourier Transform Infrared (FTIR) spectra of non-imprinted (**N**) and imprinted (**M1-M4**) monoliths S-3
Figure S6. CP-MAS solid-state ^{13}C-NMR spectra of non-imprinted (**N**) and imprinted (**M1-M4**) monoliths S-4
Figure S8. ^{1}H-NMR and COSY spectra of 3'-sialyllactose (3SL) S-4
Figure S7. Optimized geometry structure of 3SL and 6SL S-4
Figure S9. ^{1}H-NMR and COSY spectra of 6'-sialyllactose (6SL) S-4
Figure S10. ^{1}H Solid-state NMR spectra of non-imprinted (**N**) monolith. S-5
Table S1. Listing of m/z of saccharide probes selected for the extracted ion chromatograms (EICs) S-5
Table S2. Nanofiber diameters of NIP and MIP monoliths S-5
Table S3. Binding parameters from binding isotherms of 3SL and 6SL with four imprinted monoliths and NIP S-5
Table S4. Chemical shifts of hydrogens on carbon 3 of the templates used S-5
Table S5. STD differences for 3SL protons adsorbed on NIP and M4 .... S-5
Table S6. STD differences for 6SL protons adsorbed on NIP and M4 .... S-5
References S-6

* Corresponding author. Phone: +46 90 7865997; e-mail: knut.irgum@umu.se

Characterization and Evaluation Procedures

Field-Emission Scanning Electron Microscopy. Freshly fractured samples were placed on adhesive carbon foils that were affixed to standard aluminum sample stubs and thereafter secured to the holders using Ted Pella (Redding, CA, USA) conductive adhesive. Following this, a 10 nm platinum layer was coated onto all samples using a Quorum Q150TS sputter coater (Quorum Technologies, Ringmer, UK). The platinum-coated samples were subsequently investigated using a Zeiss Merlin field emission scanning electron microscope (Carl Zeiss Microscopy, Oberkochen, Germany), operated at an acceleration voltage of 5 kV. Images were captured from randomly selected areas at a set of standardized magnifications. The sizes of the fused particles and the nanofibers of the monoliths were estimated by the "Measure" function of ImageJ 1.52a[S2] using 20 and 30 measurements, respectively, for each sample.

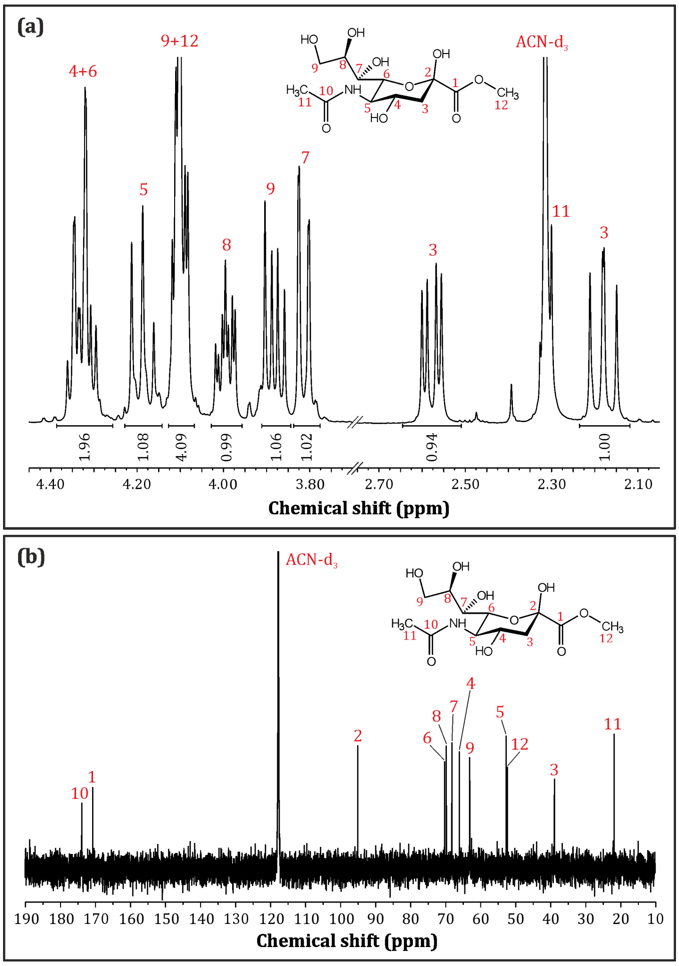

Figure S1. (a) ^{1}H and (b) ^{13}C NMR spectra of *N*-Acetylneuraminic acid methyl ester (Neu5Ac-M).

Nitrogen Cryosorption. Monolith cubes sectioned by a razor blade to cubes with side lengths of ≈ 2 mm were Soxhlet extracted overnight with methanol and thereafter dried at 60 °C in a vacuum oven at ≈ 1 kPa partial vacuum. Portions (50-150 mg) of these were transferred to dry sample tubes with an inner diameter of 9.5 mm and further dried for 2 hours at 60 °C in a Micromeritics (Atlanta, GA, USA) SmartPrep degassing unit using a stream of dry nitrogen. The samples were then directly mounted in a Micromeritics TriStar 3000 gas adsorption analyzer for multipoint adsorption-desorption analysis with nitrogen at cryoscopic temperature. The specific surface areas were determined using the Brunauer–Emmett–Teller[S1] (BET) model based on adsorption volumes in the relative pressure range of 0.18 to 0.35. The total pore volumes, average mesopore diameters ranging from 1.7 to 300 nm, and pore size distribution were estimated using the Barrett–Joyner–Halenda[S3] (BJH) scheme, based on the desorption branches of the cryosorption isotherms.

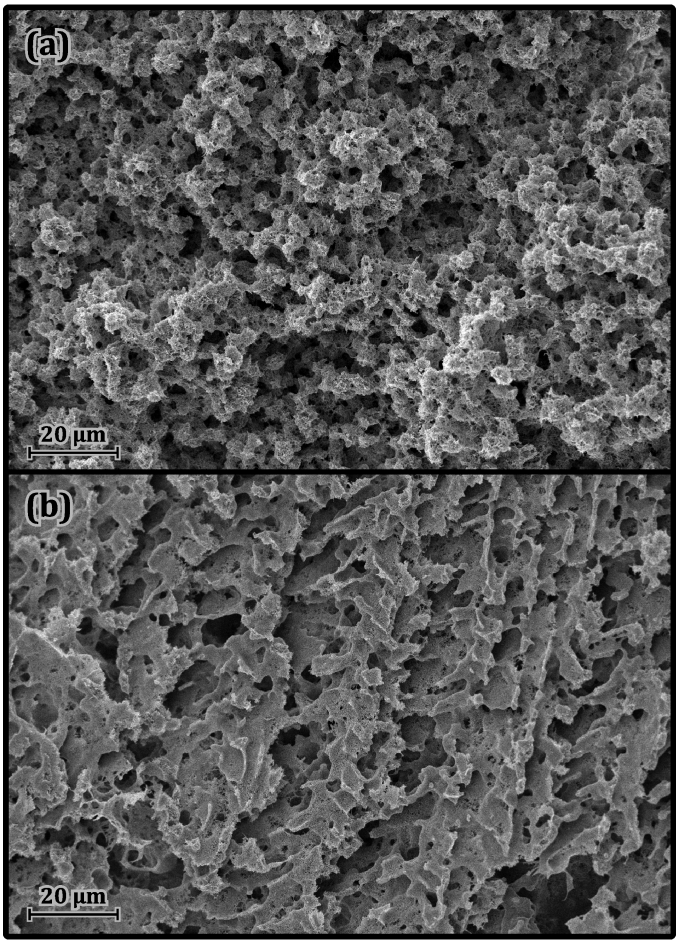

Figure S2. Field emission scanning electron micrographs of random fracture surfaces of NIP monoliths prepared by (a) slow freezing at −20 °C for 96 h, and (b) flash freezing at −196 °C for 30 s followed by curing for 96 h at −20 °C, shown at 2000x magnification. Contrast has been adjusted to represent the full 8-bit greyscale.

Diffuse Reflectance Fourier Transform Infrared Spectroscopy. Diffuse Reflectance Fourier Transform Infrared Spectroscopic measurements were conducted with an IFS 66 FTIR spectrometer from Bruker (Ettlingen, Germany), operated under partial vacuum of ≈ 400 Pa. Approximately 10 mg samples of dried monolith were manually ground together with ≈ 390 mg KBr using an agate mortar and pestle. The resulting mixture was thereafter directly transferred to a DRA-2CI diffuse reflectance cell manufactured by Harrick Scientific Products (Pleasantville, NY, USA). The spectra were recorded by co-adding 256 interferogram scans to ensure an acceptable signal-to-noise ratio. These co-added scans were then transformed to obtain spectra ranging from 4000 to 400 cm^{-1} at a spectral resolution of 4 cm^{-1}.

Liquid Nuclear Magnetic Resonance Spectroscopy. Spectral acquisitions were carried out using a Bruker AVIII 400 MHz spectrometer equipped with a 5 mm SmartProbe BBF-H/D. The 3SL and 6SL (6.00 ± 0.05 mg each) were dissolved in separate 500 µL aliquots of a 44:55 (v/v) mixture of acetonitrile-d3 and deuterium oxide. Spectra acquired at 298 K were referenced using the acetonitrile proton peak at 1.94 ppm. The **H**DO proton peaks appeared at 4.65 ppm with 3SL and at 4.75 ppm with 6SL.

Solid-state Nuclear Magnetic Resonance Spectroscopy. The ground monolith powder was transferred to zirconium oxide NMR

rotors with 4 mm inner diameter, fitted with Kel-F inserts. The ^{13}C cross-polarization magic angle spinning NMR (^{13}C CP-MAS NMR) analysis[S4] was conducted at a temperature of 298 K using a Bruker Avance III 500 MHz spectrometer with a ^{13}C CP-MAS probe spinning the sample at a rate of 8.5 kHz. The experimental procedure involved a 2.85 µs proton 90° pulse followed by a cross-polarization step using a ^{13}C spin lock field strength of 62.5 kHz. During this step, the ^{1}H field strength was ramped from 43 to 86 kHz over a duration of 1.5 ms. The 1H decoupling was performed using the SPINAL64 sequence, utilizing a decoupling field strength of 88 kHz for a duration of 6.8 ms. The FID signals were collected with a relaxation delay of 2 s and 3000 scans. Prior to Fourier transform, the accumulated FID signals were multiplied by a Gaussian apodization function and underwent manual phase and baseline correction. For chemical shift referencing, adamantane was used as an external reference, with the CH_2 signals set to 38.5 ppm. All the spectral processing steps were carried out using Bruker TopSpin 4.0.6 software.

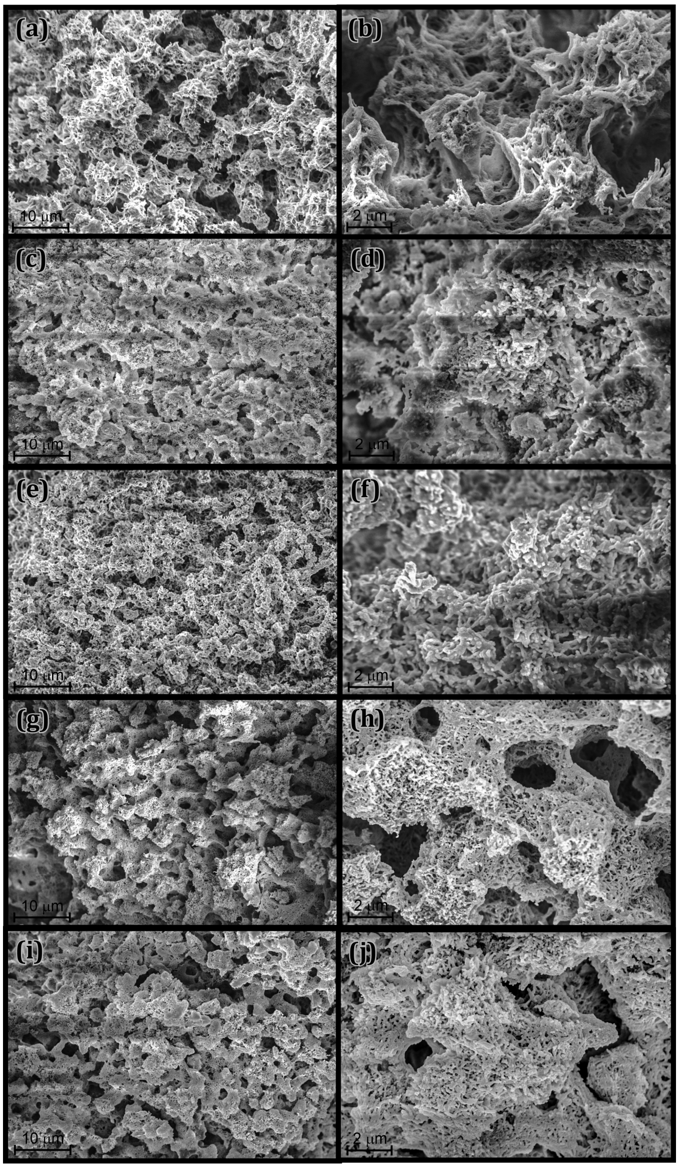

Figure S3. Field emission scanning electron micrographs (FE-SEM) of random fracture cross-section surfaces of (a, b) **N** material, and other MIP ones (c, d) **M1,** (e, f) **M2,** (g, h) **M3,** (i, j) **M4** at two different magnifications: (left) 5000, and (right) 20000. Contrast has been adjusted to represent the full 8-bit greyscale.

Geometry Structure Optimization. The structure files of the 3SL and 6SL molecules were prepared using MarvinSketch 21.13 (ChemAxon, Budapest, Hungary). The structure geometries were optimized using “Geometry Optimization” function of Molecular Operating Environment (MOE) version 2020.09 (Chemical Computing Group, Montreal, Quebec, Canada). The force field used was Amber and the model was based on adding 1 wt-% of 3SL or 6SL to acetonitrile:water 45:55 (v/v) as solvent. Other parameters were left at the default settings. The structures optimized in MOE were rendered using CCDC Mercury version 3.10.3 (Cambridge Crystallographic Data Centre, Cambridge, UK).

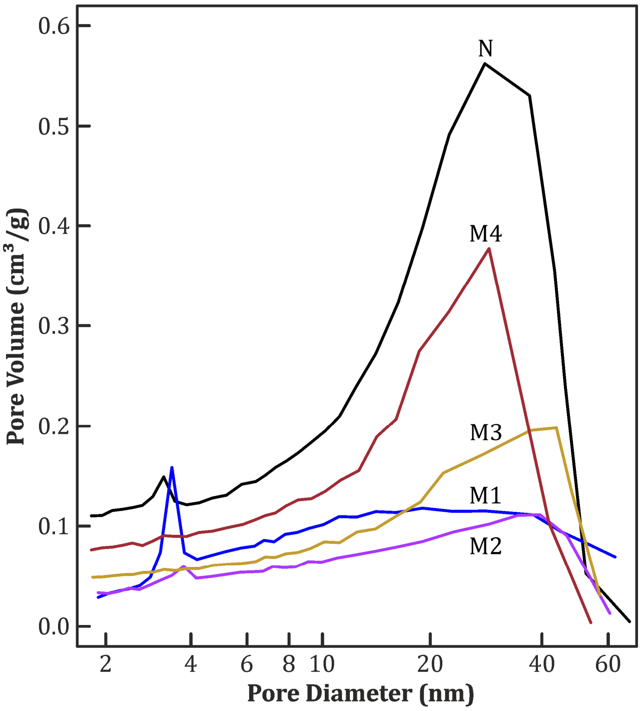

Figure S4. Plots of dV/dlog(D) pore volumes against the pore diameters of the monoliths determined from the cryosorption tests according to the Barrett–Joyner–Halenda scheme.[S2]

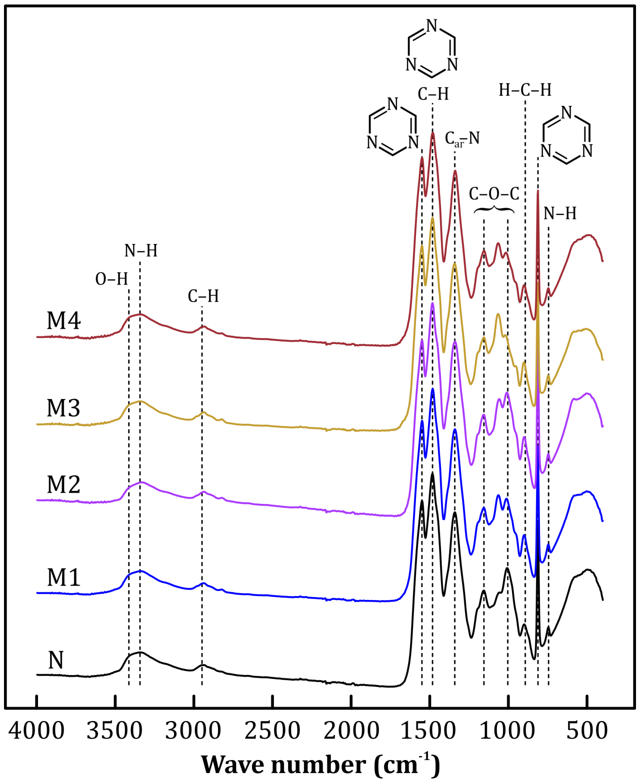

Figure S5. Fourier Transform Infrared (FTIR) spectra of non-imprinted (**N**) and imprinted (**M1**-**M4**) monoliths.

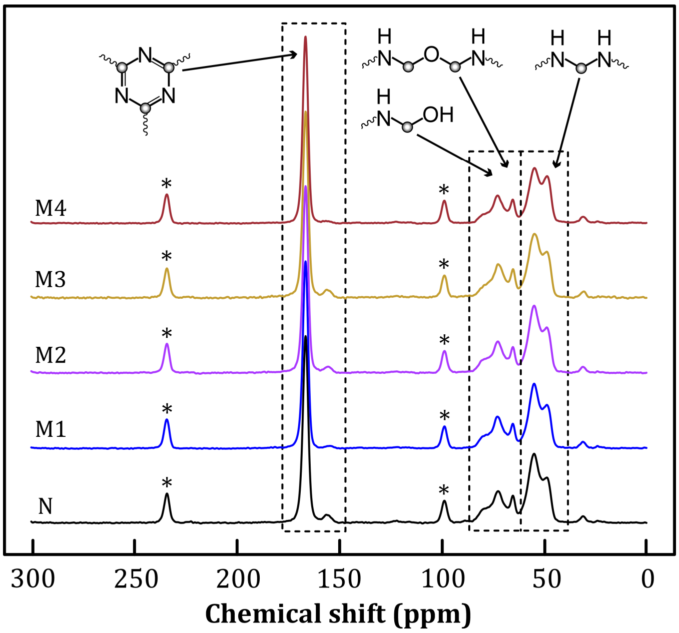

Figure S6. Cross-Polarization Magic-Angle-Spinning solid-state ^{13}C-NMR (CP-MAS) spectra of non-imprinted (**N**) and imprinted (**M1-M4**) monoliths. * Asterisks denote rotational sidebands at 8.5 kHz.

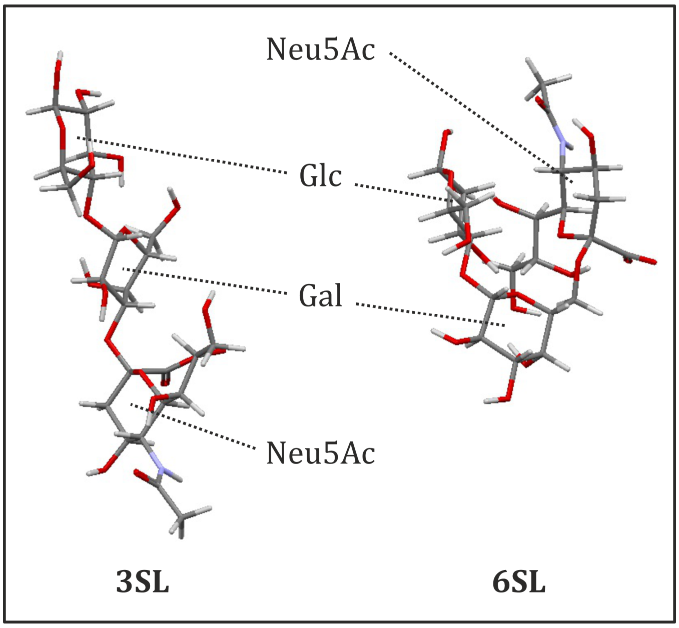

Figure S7. Geometry optimized structures of 3SL and 6SL. Optimizations were accomplished in the MOE software with acetonitrile:water mixture as medium with Sialyllactose:Acetonitrile:Water ratio 1:45:55.

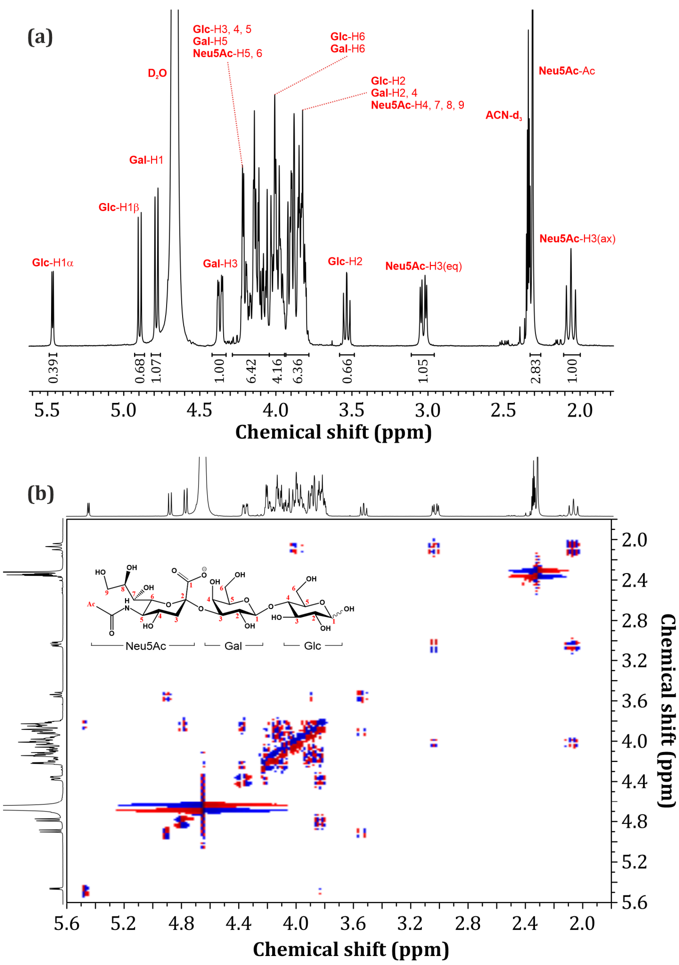

Figure S8. (a) ^{1}H-NMR and (b) COSY spectra of 3'-sialyllactose (3SL) in a mixture of acetonitrile-d3:D_2O (45:55 % v/v).

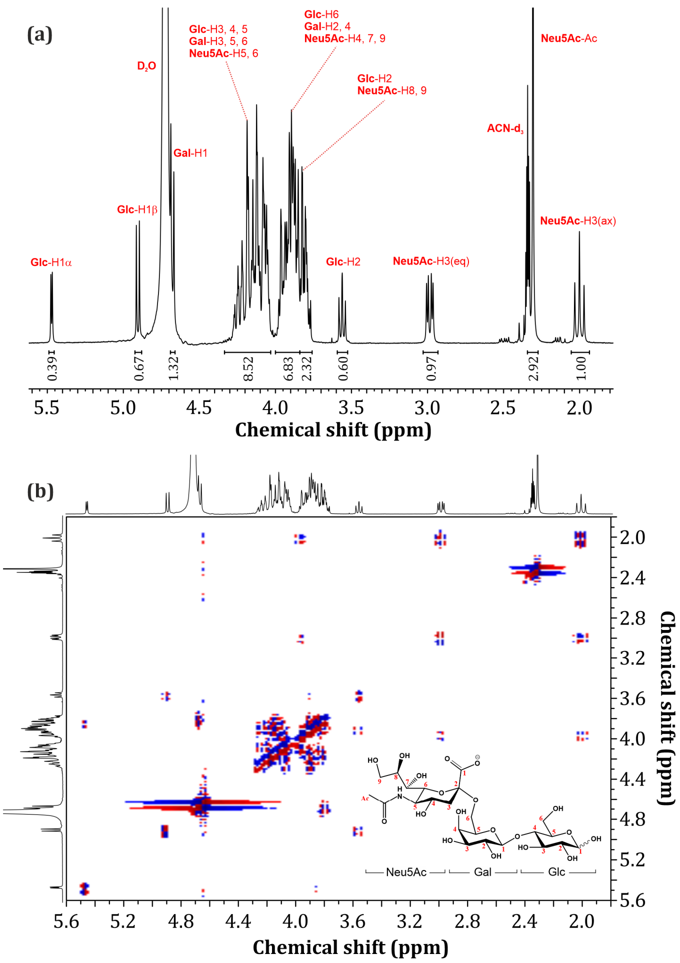

Figure S9. (a) ^{1}H-NMR and (b) COSY spectra of 6'-sialyllactose (6SL) in a mixture of acetonitrile-d3:D_2O (45:55 % v/v).

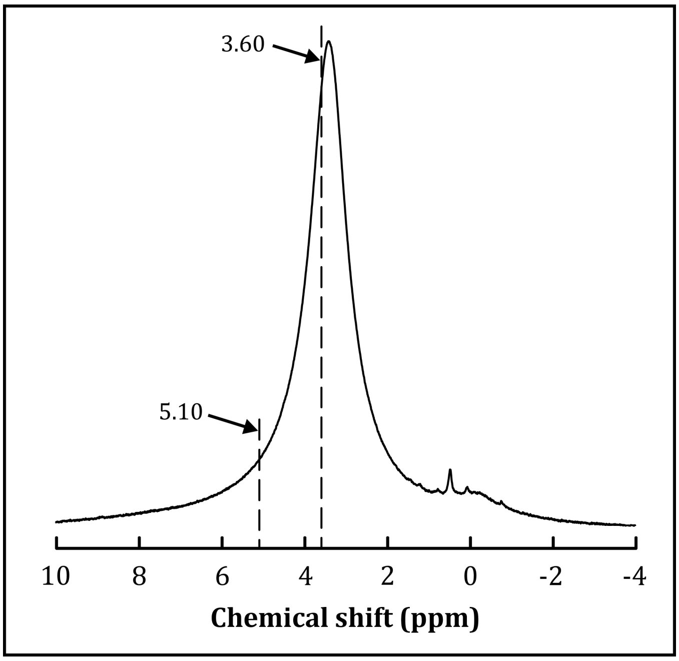

Figure S10. ^{1}H Solid-state NMR spectra of a non-imprinted (**N**) monolith. The dashed lines and associated arrows indicate the correlated chemical shifts of the saturation excitation pulses.

Table S1. Listing of m/z of saccharide probes selected for the extracted ion chromatograms.

**Compound**	**m/z**
Glucose (Glc)	179; 225
Galactose (Gal)	179; 225
Glucuronic acid (GA)	193
Lactose (Lac)	387
3' Sialyllactose (3SL)	632.5
6' Sialyllactose (6SL)	632.5

Table S2. Nanofiber diameters of NIP and MIP monoliths.

**Monolith**	**Nanofiber diameter (nm)**
N	36.3 ± 6.9
M1	125 ± 27
M2	123 ± 12
M3	59 ± 11
M4	68.4 ± 7.5

Values are based on measurement of 30 segments in FE-SEM images.

Table S3. Binding parameters from binding isotherms of 3SL and 6SL with four imprinted monoliths and NIP.

**Monolith**	**Capacity** (μmol/m^2)		**Affinity constant** (mM^{-1})	
	3SL	6SL	3SL	6SL
N	1.09 ± 0.031	0.60 ± 0.057	4.64 ± 0.23	0.75 ± 0.11
M1	1.38 ± 0.34	0.84 ± 0.064	0.74 ± 0.28	1.41 ± 0.21
M2	1.17 ± 0.18	1.17 ± 0.069	2.04 ± 0.69	1.97 ± 0.25
M3	1.25 ± 0.21	1.14 ± 0.34	7.9 ± 3.4	1.34 ± 0.65
M4	1.14 ± 0.13	1.32 ± 0.15	5.7 ± 1.4	1.86 ± 0.38

Table S4. Chemical shifts of hydrogens on carbon 3 of the templates used.

**Templates**	**α-anomer**		**β-anomer**	
	H3eq	H3ax	H3eq	H3ax
Neu5Ac	2.99 (2)	1.93 (3)	2.58 (46)	2.13 (49)
Neu5Ac-M	2.98 (2)	1.94 (3)	2.59 (46)	2.16 (49)
3SL	3.00 (49)	2.02 (51)	-	-
6SL	2.99 (49)	1.98 (51)	-	-

Values in parentheses are signal percentages for each template using 44:55 (v/v) mixture of acetonitrile d3 and deuterium oxide as solvent. ax = axial, eq = equatorial

Table S5. STD differences for 3SL protons adsorbed on NIP and M4 materials at saturation frequencies 1800 and 2550 Hz with off-resonance frequency at 12600 Hz.

**Proton**	**Chemical shift** (ppm)	**STD difference** (%)	
		1800 Hz	2550Hz
**Glc**-H1α	5.44	0	0
**Glc**-H1β	WPD[a)]	–	–
**Gal**-H1	WPO[b)]	–	–
**Gal**-H3	4.33	20	2.5
**Glc**-H3, 4, 5<br>**Gal**-H5<br>**Neu5Ac**-H5, 6	4.25 – 4.01	10	-3.4
**Glc**-H6<br>**Gal**-H6	4.01 – 3.91	13	46
**Glc**-H2<br>**Gal**-H2, 4<br>**Neu5Ac**-H4, 7, 8, 9	3.91 – 3.65	−10	2.2
**Neu5Ac**-H3(eq)	3.00	14	−11
**Neu5Ac**-Ac	2.28	22	25
**Neu5Ac**-H3(ax)	2.02	0.5	−6.0

a) WPD, Water peak disturbance; b) WPO, Water peak overlap.

Table S6. STD differences for 6SL protons adsorbed on NIP and M4 materials at saturation frequencies 1800 and 2550 Hz with off-resonance frequency at 12600 Hz.

**Proton**	**Chemical shift** (ppm)	**STD difference** (%)	
		1800 Hz	2550Hz
**Glc**-H1α	5.47	0	0
**Glc**-H1β	WPD[a)]	–	–
**Gal**-H1	WPO[b)]	–	–
**Glc**-H3, 4, 5<br>**Gal**-H3, 5, 6<br>**Neu5Ac**-H5, 6	4.31 – 4.01	20	4.0
**Glc**-H6<br>**Gal**-H2, 4<br>**Neu5Ac**-H4, 7, 9	4.01 – 3.83	21	6.0
**Glc**-H2<br>**Neu5Ac**-H8, 9	3.83 – 3.72	32	51
**Neu5Ac**-H3(eq)	2.99	−2.4	−25
**Neu5Ac**-Ac	2.31	44	45
**Neu5Ac**-H3(ax)	1.98	−11	−9.4

a) WPD, Water peak disturbance; b) WPO, Water peak overlap.

References

(S1) Brunauer, S.; Emmett, P. H.; Teller, E. Adsorption of Gases in Multimolecular Layers. *J. Am. Chem. Soc.* **1938**, *60*, 309–319.

(S2) Schneider, C. A.; Rasband, W. S.; Eliceiri, K. W. NIH Image to ImageJ: 25 years of image analysis. *Nature Methods* **2012**, *9*, 671–675.

(S3) Barrett, E. P.; Joyner, L. G.; Halenda, P. P. The Determination of Pore Volume and Area Distributions in Porous Substances. I. Computations from Nitrogen Isotherms. *J. Am. Chem. Soc.* **1951**, *73*, 373–380.

(S4) Fung, B. M.; Khitrin, A. K.; Ermolaev, K. An improved broadband decoupling sequence for liquid crystals and solids. *J. Magn. Reson.* **2000**, *142*, 97-101.
